# Retained Foreign Body in Transplanted Liver

**Published:** 2015-11-01

**Authors:** C. Kayaalp, S. Kırmızı, R. Kutlu, M. A. Yagci, B. Isik, S. Yilmaz

**Affiliations:** Inonu University, Liver Transplantation Institute, Malatya, Turkey

**Keywords:** Liver transplantation, Foreign body, Post-operative complication, Portal vein

## Abstract

Liver transplantation is a technically complex and long surgical procedure. A large quantity of various materials such as catheters, sutures, needles and clips are frequently used during the procedure. These materials may enter in the liver from the vascular or biliary orifices inadvertently. A 50-year-old patient who had hepatic failure due to HBV underwent a deceased-donor liver transplantation. The deceased donor was a 75-year-old HbsAg^+^ man. The recipient had subfebrile fever and leukocytosis post-operatively. A control computed tomography revealed a cuneiform ischemic area, and a foreign body inside the right anterior portal vein branch proximal to this ischemic region. A 10-F Nelaton catheter, 5-cm long, was removed from the portal vein by surgery. Retrospectively, we understood that the portal vein was cut during the back-table procedure and the portal vein catheter was replaced with a larger one for better irrigation. Most probably, the original catheter was cut together with the portal vein, and the tip of the catheter was retained in the portal system and migrated into the liver. As far as we know, such a complication of liver transplantation has never been described previously.

## INTRODUCTION

It is a routine procedure worldwide to count the surgical materials and instruments before closing the abdomen. Despite all these precautions, some pieces may be left in the body cavities [1-7]. Retained foreign body cases are estimated to be seen in one of 1000–1500 patients who underwent laparotomy [8, 9]. Here, we report on an extraordinary way of retaining a foreign body at surgery.

## CASE REPORT

**Figure 1:**
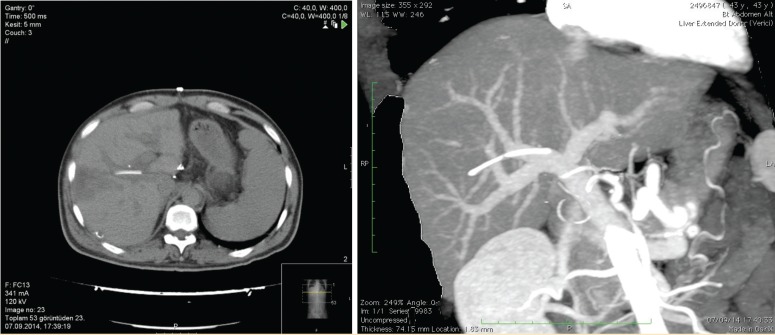
Computed tomography revealing a thin, long foreign body inside the anterior branch of the portal vein supplying this lobe

**Figure 2:**
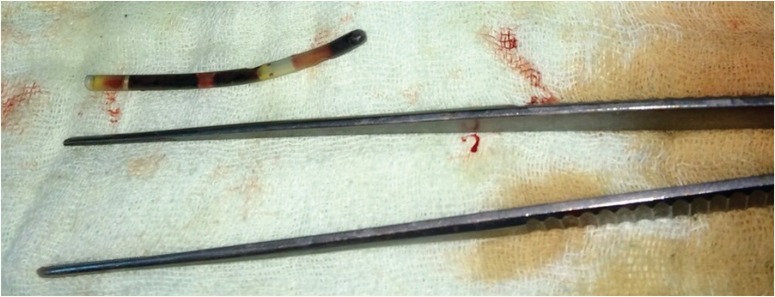
The foreign body removed from the anterior branch of the right portal vein

Brain death occurred due to subarachnoid hemorrhage in a 75-year-old, HbsAg^+^ donor in another transplant center and the local transplant center did not accept this marginal graft. Local transplant team harvested the graft and sent it to our center within nine hours. Back-table procedure was performed in our center and the graft was transplanted to a 50-year-old, HbsAg^+^ patient who had chronic hepatic failure. There were no intra-operative complications. In the post-operative period, however, an increase in leukocyte count and fever were seen. Abdominal computed tomography on the 9^th^ day revealed a cuneiform ischemic area in the right liver lobe. There was a thin, long foreign body inside the anterior branch of the portal vein supplying this lobe (Fig 1). Removal of the catheter by interventional radiology was though but found more difficult than its surgical removal. Re-laparotomy on day 10 revealed the ischemic area in the right lobe. The portal vein was opened from an extra-anastomotic area in a transverse plane under total hepatic vascular occlusion and a 10-F catheter piece, 5-cm long, was removed using a clamp from the anterior branch of the right portal vein (Fig 2). The portal vein was closed primarily. The fever subsided and the leukocyte count was normalized. There were no complication and the patient was discharged on the 18^th^ day uneventfully. A control computed tomography on day 40 revealed that the necrotic area had regressed. 

Retrospectively, we understood that the graft portal vein was cut during the back-table procedure and the portal vein catheter was replaced with a larger one for better irrigation. Most probably, the original catheter was cut together with the portal vein, and the tip of the catheter was retained in the portal system and migrated into the liver.

## DISCUSSION

Inadvertently retained foreign body would cause significant problems for both patients and health care professionals. It causes morbidity and mortality risk for patients and malpractice lawsuits against health care professionals. Most of the retained foreign bodies consist of surgical gauzes and surgical instruments [8, 9]. 

Liver transplantation is a multi-staged complex operation including donor hepatectomy, back-table procedure, recipient hepatectomy and implantation. This operation takes more time compared to other surgical operations; many medical staffs participate in the procedure. In the literature, there are some cases where sutures, clips, stents or catheters retained particularly in the bile ducts; all these remnants are usually forgotten during the implantation of the new liver [10]. However, to the best of our knowledge, transplantation of a forgotten foreign body in a transplanted graft has not been reported yet. Moreover, a foreign body in a portal vein has been never reported to date.

In the case of a suspected foreign body, intra-operative imaging modalities are commonly used. Furthermore, novel techniques such as barcode scanning and radio-frequency identification have not been used widely [1]. A recent approach concerning precautions taken into account to prevent retained foreign bodies, is based on counting all the materials used in the surgery [1-7]. However, with a success rate of 88%, this method may not prevent all the complications [8]. It should be remembered that in addition to counting the materials and instruments used, checking the completeness of these materials and instruments is of paramount importance. This procedure should be done strictly during harvesting and back-table procedure as well. 

It is advised to extract all foreign materials including irrigation catheters from the donated graft before transporting from a center to another one: first to prevent anyiatrogenic trauma especially to the endothelium and second to prohibit losing the catheters inside the graft.
